# Metagenomic strain detection with SameStr: identification of a persisting core gut microbiota transferable by fecal transplantation

**DOI:** 10.1186/s40168-022-01251-w

**Published:** 2022-03-25

**Authors:** Daniel Podlesny, Cesar Arze, Elisabeth Dörner, Sandeep Verma, Sudhir Dutta, Jens Walter, W. Florian Fricke

**Affiliations:** 1 Department of Microbiome Research and Applied Bioinformatics, University of Hohenheim, Stuttgart, Germany; 2 Current address: Ring Therapeutics, Cambridge, MA USA; 3 Division of Gastroenterology, Sinai Hospital of Baltimore, Baltimore, MD USA; 4 APC Microbiome Ireland, School of Microbiology, and Department of Medicine, University College Cork, Cork, Ireland; 5 Institute for Genome Sciences, University of Maryland School of Medicine, Baltimore, MD USA

## Abstract

**Background:**

The understanding of how microbiomes assemble, function, and evolve requires metagenomic tools that can resolve microbiota compositions at the strain level. However, the identification and tracking of microbial strains in fecal metagenomes is challenging and available tools variably classify subspecies lineages, which affects their applicability to infer microbial persistence and transfer.

**Results:**

We introduce SameStr, a bioinformatic tool that identifies shared strains in metagenomes by determining single-nucleotide variants (SNV) in species-specific marker genes, which are compared based on a maximum variant profile similarity. We validated SameStr on mock strain populations, available human fecal metagenomes from healthy individuals and newly generated data from recurrent *Clostridioides difficile* infection (rCDI) patients treated with fecal microbiota transplantation (FMT). SameStr demonstrated enhanced sensitivity to detect shared dominant and subdominant strains in related samples (where strain persistence or transfer would be expected) when compared to other tools, while being robust against false-positive shared strain calls between unrelated samples (where neither strain persistence nor transfer would be expected). We applied SameStr to identify strains that are stably maintained in fecal microbiomes of healthy adults over time (strain persistence) and that successfully engraft in rCDI patients after FMT (strain engraftment). Taxonomy-dependent strain persistence and engraftment frequencies were positively correlated, indicating that a specific core microbiota of intestinal species is adapted to be competitive both in healthy microbiomes and during post-FMT microbiome assembly. We explored other use cases for strain-level microbiota profiling, as a metagenomics quality control measure and to identify individuals based on the persisting core gut microbiota.

**Conclusion:**

SameStr provides for a robust identification of shared strains in metagenomic sequence data with sufficient specificity and sensitivity to examine strain persistence, transfer, and engraftment in human fecal microbiomes. Our findings identify a persisting healthy adult core gut microbiota, which should be further studied to shed light on microbiota contributions to chronic diseases.

**Video abstract**

**Supplementary Information:**

The online version contains supplementary material available at 10.1186/s40168-022-01251-w.

## Background

Disturbances of the human gut ecosystem have been implicated in many metabolic, inflammatory, and infectious diseases, based on altered taxonomic or functional microbiota compositions in affected individuals. However, attempts to identify consistent, disease-specific microbiome markers have been less successful, as reported associations vary and have frequently not been consistent between studies [1, 2]. Among many other factors [3], taxonomic and functional variations between microbial subspecies or strains that are members of the human microbiome [4] can produce inconsistent findings, but have not been comprehensively characterized. The species *Ruminococcus gnavus*, for example, has been linked to inflammatory bowel diseases [5], but disease associations appear to be specific to only one of two described subspecies clades [6] and may be dependent on strain-specific variations in carbohydrate utilization [7] or pro-inflammatory polysaccharide production [8], emphasizing the need for health-related microbiome studies to focus at subspecies level microbiota variations. Moreover, many of the ecological forces that shape microbiomes in health and disease, or after perturbation and therapeutic modulation, involve microbial interactions, such as competition, inhibition, or predation, which can be strain-dependent [9–11] and require compositional microbiota analyses to provide strain-level taxonomic resolution.

Shotgun metagenomics has the potential for a maximum phylogenetic resolution that can theoretically resolve even individual microbial genomes in a metagenomic sample [12]. Consequently, several bioinformatic methods have been introduced to identify microbial strains in metagenomes, based on the generation of metagenome-assembled genomes (MAGs, see [13], Strainberry [12], and STRONG [14]) or the mapping of individual metagenomic reads to universal (see StrainFinder [15] and mOTUs2 [16]) or taxon-specific marker genes (see StrainPhlAn [17]), or whole-genomes (see InStrain [18]) to detect phylogenetically informative, strain-specific, single nucleotide variant (SNV) profiles. Microbiota strain profiling has been successfully applied to study strain-specific adaptations to human body sites [4]; associations with individual human hosts, families, and geography [17, 19]; and transmission along the gastrointestinal tract [20], from mothers to infants [21–23] and from the donors to the recipients of fecal microbiota transplantation (FMT) [15, 24, 25]. Yet, strain-level microbiota analysis is hampered by inconsistent “strain” definitions [26, 27] and available methods exhibit variable sensitivities and specificities, which have not been comprehensively compared and validated. For example, the taxonomic classification of strains based on universal marker gene phylogenetic comparisons can produce inconsistent assignments relative to established taxonomies [15, 24]. Detection may also be limited to the dominant strain in a metagenomic sample [17] or depend on the availability of completely sequenced reference genomes for comparison [28] Finally, non-stringent similarity thresholds can result in distinct subspecies lineages to become assigned to the same strain, which is problematic if the strain is used to infer microbial persistence or transfer. In this case, for example, human intestinal microbiomes may contain the same “strain,” i.e., a subspecies lineage with widespread prevalence in the human population, without having experienced direct microbial transfer.

To address these limitations, we developed SameStr as a new tool for the detection of shared strains in metagenomic samples. SameStr leverages the StrainPhlAn approach to map metagenomic reads to clade-specific marker genes [17], which compared to other tools affords increased taxonomic resolution [29]. However, SameStr extends the detection of shared strains to subdominant members of multi-strain species populations. This is achieved by considering multiple alleles instead of the consensus sequence at polymorphic positions in the metagenomic marker gene alignments. We validated SameStr using new and available metagenomes, including temporally linked sample pairs (i.e., collected from the same individual at different time points) or physically linked sample pairs (i.e., collected from different, connected individuals, such as FMT donors and recipients). We demonstrate increased sensitivity for the detection of subdominant shared strains and increased specificity for the detection of species-specific strains, which are not shared between unrelated sample pairs, over previous methods. We applied SameStr to identify a core gut microbiota of strains that persist over time in healthy adults and to determine the contributions of recipient- and donor-derived strains to the post-FMT patient microbiota, illustrating SameStr’s utility to study microbiome stability and transfer across different settings. We further show that persisting strains in healthy adults frequently belonged to the same species as donor-derived strains in post-FMT patients, suggesting the existence of a healthy adult core gut microbiota that is transferable from donors to rCDI patients by FMT.

## Results

### Detection of shared strains in metagenomic samples with SameStr

**Fig. 1:**
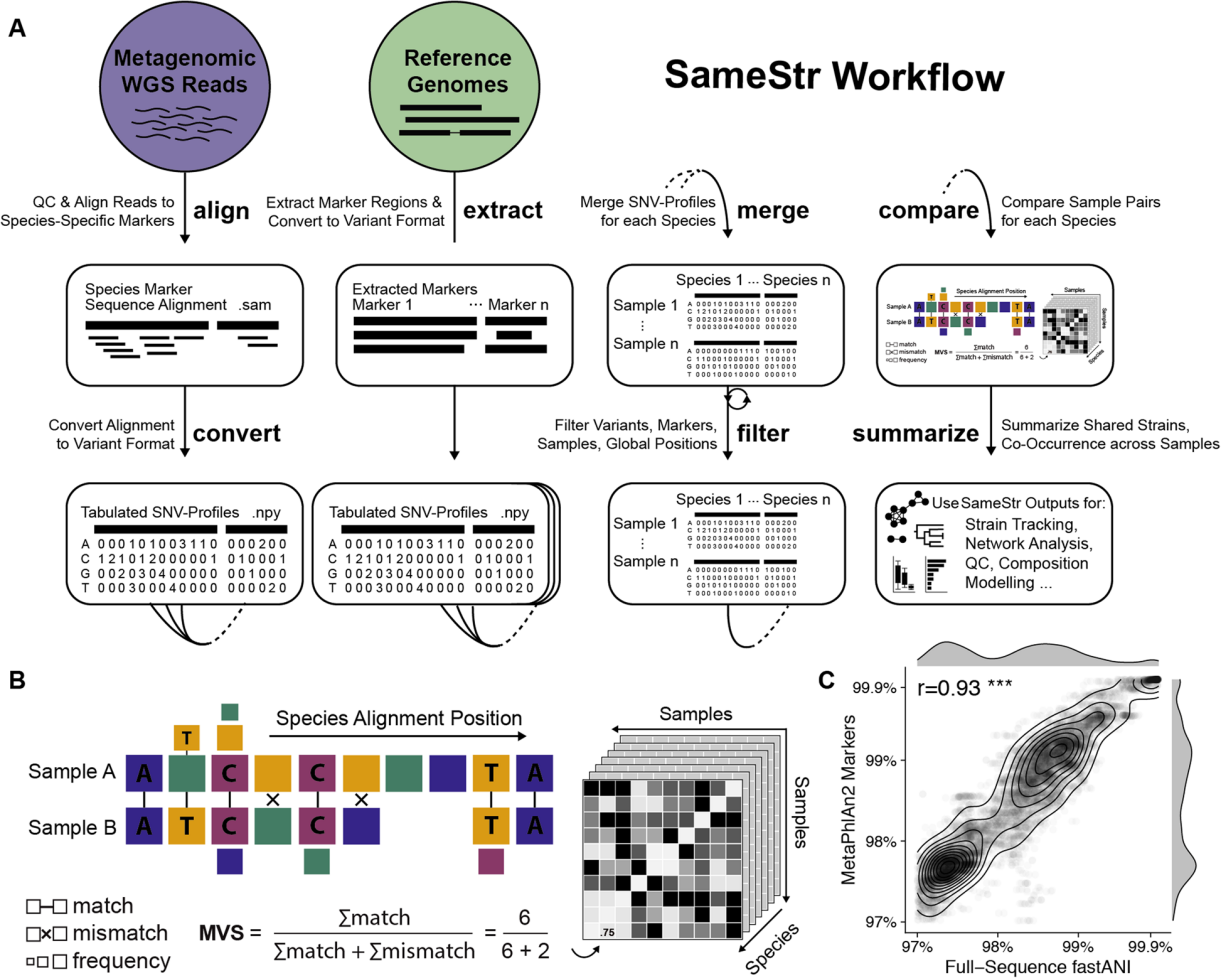
Species-specific shared strain detection in metagenomic samples with SameStr. **A** Schematic of the SameStr workflow. SameStr has been implemented modularly, including optional wrapper functions for quality preprocessing and alignment of whole-genome shotgun (WGS) metagenomic reads to species-specific MetaPhlAn markers (align), functions for the conversion to nucleotide variant profiles (convert), extraction of markers from genome sequences (extract), sample and reference pooling (merge), extensive global, per-sample, marker and position filtering (filter) and comparison of SNV profiles (compare) based on maximum variant similarity (MVS). SameStr outputs (summarize) tables denoting pairwise comparison results, including species alignment similarity and overlap, and co-occurrence of taxa at distinct taxonomic levels (based on MetaPhlAn) and at the strain level. **B** SameStr identifies shared strains in metagenomic samples by calculating a pairwise MVS, using all single-nucleotide variants detected in the read alignments of these samples to species-specific marker genes. **C** To assess the MetaPhlAn-based phylogenetic resolution (db_v20) and validate the 99.9% similarity threshold of shared strains, which is used by SameStr, 458 bacterial genomes from 20 of the most abundant and prevalent fecal microbiota species in our rCDI cohort (Table S4) were compared with MetaPhlAn2 [30] and based on average nucleotide identities (ANIs) as determined with FastANI [31]. MetaPhlAn2 and FastANI-based pairwise sequence similarities are strongly correlated (Spearman’s *r* = 0.93, *p* < 2.2e−16, *n* = 9813), demonstrating comparable phylogenetic resolution. Genome similarities exhibit a multimodal distribution (two-dimensional density kernel contours): reference genomes share peak sequence similarities at 97.5%, 99.0%, and above 99.9% identity that reflect the presence of distinct species, subspecies, and strains in the reference dataset

We developed the SameStr tool based on a workflow related to StrainPhlAn [17] to identify shared microbial strains in distinct metagenomic samples using within-species phylogenetic sequence variations (Fig. 1A). In brief, metagenomic input data are first quality-filtered and trimmed to reduce sequencing errors and then mapped to the MetaPhlAn reference database of species-specific marker genes [30], in order to limit the interference of higher-level taxonomic sequence variations with strain detection. Individual alignments for each sample and species are filtered and merged. Strains shared between samples are identified by comparing alignments, using a maximum variant profile similarity (MVS), which is calculated as the fraction of identical nucleotide positions in both alignments divided by the total length of the shared alignment (Fig. 1B). A comparison of SameStr’s resource requirements (total CPU time, CPU time per sample, and average RAM use), compared to metagenomic sequence preprocessing with Kneaddata and taxonomic analysis with MetaPhlAn is shown in the supplement (Fig. S1). In contrast to StrainPhlAn, which determines a consensus sequence for each marker alignment and compares metagenomes based on the consensus variant similarity (CVS) that only reflects the dominant strain in each sample, SameStr considers all detected single nucleotide variants to calculate MVS, including polymorphic positions with different relative allelic frequencies (default: ≥ 10%), thereby including non-dominant strains into the sample comparison. SameStr calls shared strains in two metagenomic samples if the corresponding species alignments share a minimum overlap (default: ≥ 5 kb) and MVS (default: ≥ 99.9%) over all detected sites. A similarity threshold of 99.9% for comparing MetaPhlAn marker genes (db_v20) was previously shown to differentiate between microbial strains within species and subspecies [17, 32, 33] and is further validated by our phylogenetic comparison of reference genomes based on whole-genome average nucleotide identity (ANI) (Fig. 1C).

### Validation of sensitivity and specificity of SameStr in comparison to other strain prediction tools

We first evaluated SameStr’s performance on synthetic, simulated metagenomes from species containing multiple strains. Mock sequence data from 100 individual isolates from 20 frequent and abundant bacterial gut species (Table S4) were mixed in various combinations to simulate metagenomes containing species composed of multiple strains and variable complexity and sequencing depth. For each species, simulated shotgun sequence data from a reference genome (at a 5-fold sequencing depth and showing typical sequencing error profiles, see “Materials and methods”) were compared to simulated metagenomes. These included the same reference genome (showing an independent typical error profile) at variable sequencing depths (target strain coverage), combined with additional sequence data from between 1 and 4 other available genomes from the same species at varying sequencing depths (noise coverage).

**Fig. 2:**
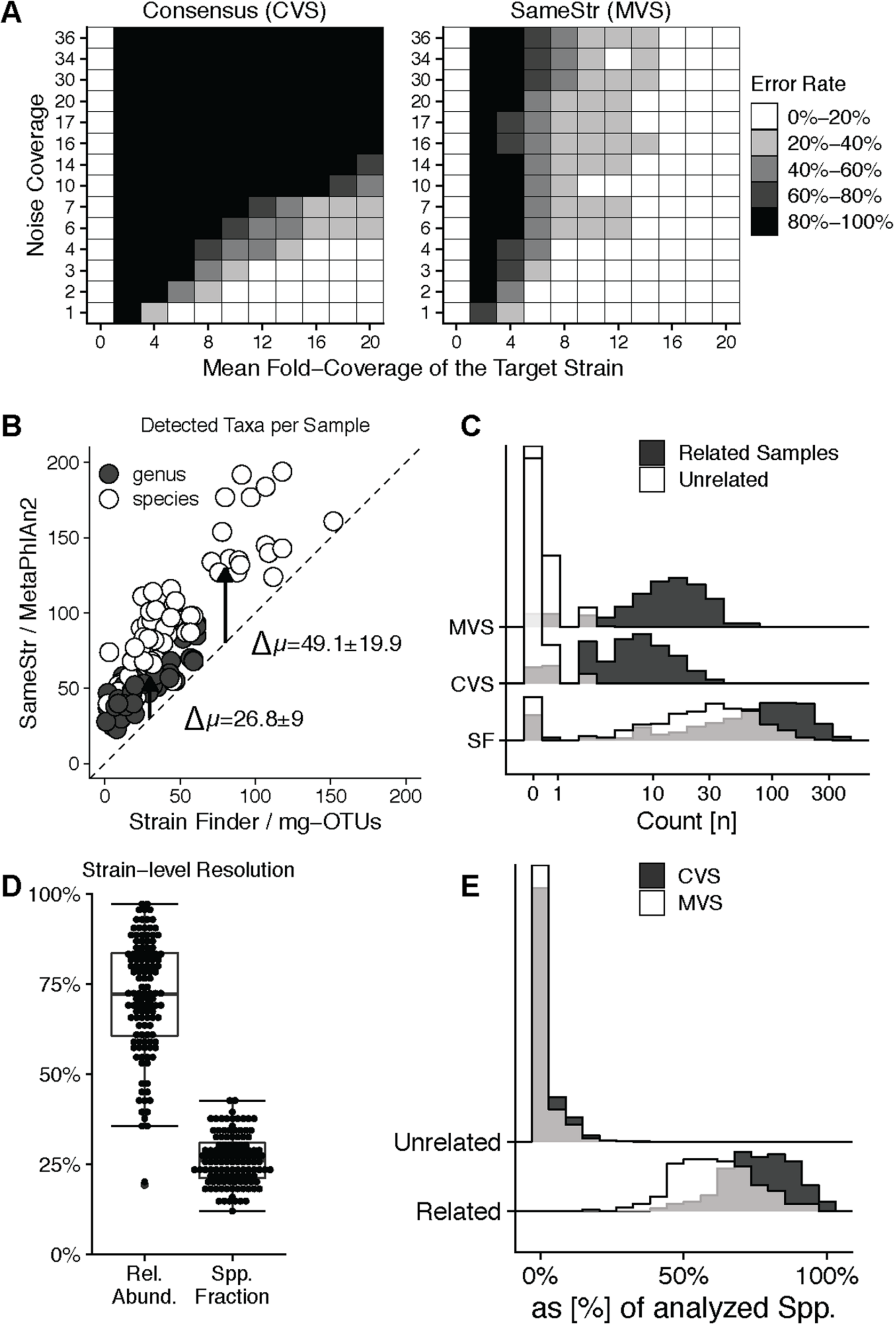
Sensitivity and specificity comparison to other strain prediction tools. **A** SameStr detects dominant and subdominant strains at low sequencing depth (mean-fold target strain coverage) and relative abundance (i.e., high noise coverage) in simulated metagenomes (*n* = 3276) of multi-strain species populations, compared to consensus variant profile similarity (CVS)-based methods. **B** Using MetaPhlAn’s clade-specific marker gene database (db_v20), SameStr identifies more genera and species per metagenomic sample (*n* = 65) than StrainFinder, which uses mg-OTUs that are defined based on phylogenetic comparisons of universally distributed bacterial genes from the AMPHORA database. **C** Fewer shared strain calls demonstrate the increased specificity of SameStr compared to StrainFinder, which allows for the differentiation of related (n=555) and unrelated (n=1,525) sample pairs. **D** Cumulative relative abundance and fraction of species for which strain-level resolution was achieved with SameStr in fecal metagenomes from a reference cohort of 67 longitudinally sampled healthy adults (*n* = 202). **E** SameStr’s MVS-based method detects shared strains in a larger fraction of species in related (same individual, *n* = 281) but not in unrelated (different individuals, *n* = 20,020) sample pairs of the control cohort (*n* = 202 individuals) compared to CVS-based methods

SameStr’s strain predictions based on maximum variant profile similarity (MVS) were compared to those of a StrainPhlAn-equivalent consensus variant similarity (CVS)-based approach across a total of 3276 simulated combinations (Fig. 2A). SameStr outperformed the consensus-based approach for the detection of dominant target strains (≥ 50% relative strain abundance at ≥ 5-fold target strain sequencing depth) in multi-strain species populations, detecting 85% of shared strains compared to 59% with the CVS-based approach. SameStr also detected 57% of shared strains among subdominant strains (15–50% relative strain abundance at ≥ 5-fold target strain sequencing depth), compared to only 2% for the consensus-based method. The better performance of SameStr compared to consensus-based methods in even the identification of dominant strains might be due to the lower sensitivity of the MVS-based approach to sequencing errors and wrong consensus calls at polymorphic and/or low-coverage positions of the metagenomic read alignment. Importantly, advantages in accuracy were not accompanied by reduced specificity, as both approaches were robust against false-positive shared strain calls even in complex multi-strain species mixtures (see 0-fold target strain coverage in Fig. 2A).

The StrainFinder tool has been developed to study strain-level microbiota dynamics in the course of fecal microbiota transplantation (FMT) [15]. StrainFinder used phylogenetic comparisons of 31 widely distributed, single-copy marker genes from the AMPHORA database [34] to define metagenomic operational taxonomic units (mg-OTUs) and call distinct strains based on sequence variations within these species equivalents [15]. We compared the performances of SameStr and StrainFinder with respect to (i) taxonomic sensitivity, i.e., the number of microbial genera and species assessed for shared strain detection (Fig. 2B), and (ii) specificity for the detection of ‘unique’ shared strain events, i.e., the frequency of shared strain predictions in unrelated sample pairs, which would interfere with our goal to use shared strains to infer strain persistence or transfer (Fig. 2C). Using the published datasets and taxonomic profiles from the original StrainFinder publication [15], SameStr consistently detected more species and genera, both across the entire dataset (154 vs. 116 genera and 399 vs. 306 species/mg-OTUs) and per sample (50.54 ± 15.0 vs. 23.78 ± 16.67 genera and 97.62 ± 39.6 vs. 48.48 ± 33.88 species/mg-OTUs; values shown as mean ± sd) (Fig. 2B). Differentially detected taxa included prominent members of the gastrointestinal tract microbiota, such as *Bacteroides* spp. (6.54 ± 5.35 species vs. 3.87 ± 4.70 mg-OTUs per sample), *Clostridium* spp. (4.81 ± 4.05 species vs. 2.43 ± 3.25 mg-OTUs per sample), and *Lactobacillus* spp. (5.06 ± 3.33 species vs. 1.41 ± 2.37 mg-OTUs per sample).

For the detection of shared strains, we divided the original FMT dataset from Smillie et al. into related and unrelated sample pairs. Related sample pairs included corresponding FMT recipient and donor samples, pre and post-FMT patient samples, and distinct samples from the same donor or post-FMT patient. SameStr detected on average 14.77 (median = 12, range = 0–67) shared strains in 555 related sample pairs and 0.45 (median = 0, range = 0–8) shared strains in 1525 unrelated sample pairs. By comparison, StrainFinder reported on average 93.13 (median = 73, range = 0–384) shared strains in related but also 35.16 (median = 25, range = 0–238) shared strains in unrelated sample pairs (Fig. 2C). These findings suggest that StrainFinder classifies subspecies lineages with broader prevalence in human populations as shared strains, which based on SameStr’s more conservative definition of “unique” shared strains would be considered false-positive predictions.

To further assess SameStr’s rate of false-positive shared strain predictions in fecal metagenomes, we downloaded a reference dataset (‘control’) from the curatedMetagenomicData package [35], consisting of 202 fecal metagenomes from four different studies, including 67 healthy adults that were sampled multiple times over a period of up to 1 year (see “Materials and methods” and Table S2). On average, strain-level resolution was obtained for 26.2% ± 6.8 of species or 71.4% ± 15.9 relative abundance per sample (Fig. 2D). This control dataset was divided into related sample pairs from the same individual, which would be expected to share strains, and unrelated sample pairs from distinct individuals, which would not be expected to share strains. Compared to the consensus-based method that is used by StrainPhlAn, SameStr detected more shared strains in 281 related sample pairs (range = 4–43, median = 14) but not in 20,020 unrelated sample pairs (range = 0–4, median = 0) (Fig. 2E), demonstrating increased sensitivity without compromising the low rates of false-positive shared strain detections that both approaches showed.

In summary, SameStr can detect shared strains in synthetic and real metagenomes, including from single- and multi-strain species populations, with improved accuracy for low-abundant and subdominant strains compared to StrainPhlAn and taxonomically more accurate and restrictive predictions of shared strains compared to StrainFinder.

### Identification of strain persistence and engraftment in healthy individuals and rCDI patients after FMT

To gain insights into (i) microbiome stability in healthy individuals and (ii) microbiome transfer in the course of FMT, we applied SameStr to measure strain persistence and engraftment in our reference dataset of fecal metagenomes from healthy adult individuals and a combined FMT dataset with fecal samples from FMT-treated rCDI patients and their donors from our previously described cohort [36] and the study by Smillie et al. [15].

**Fig. 3.**
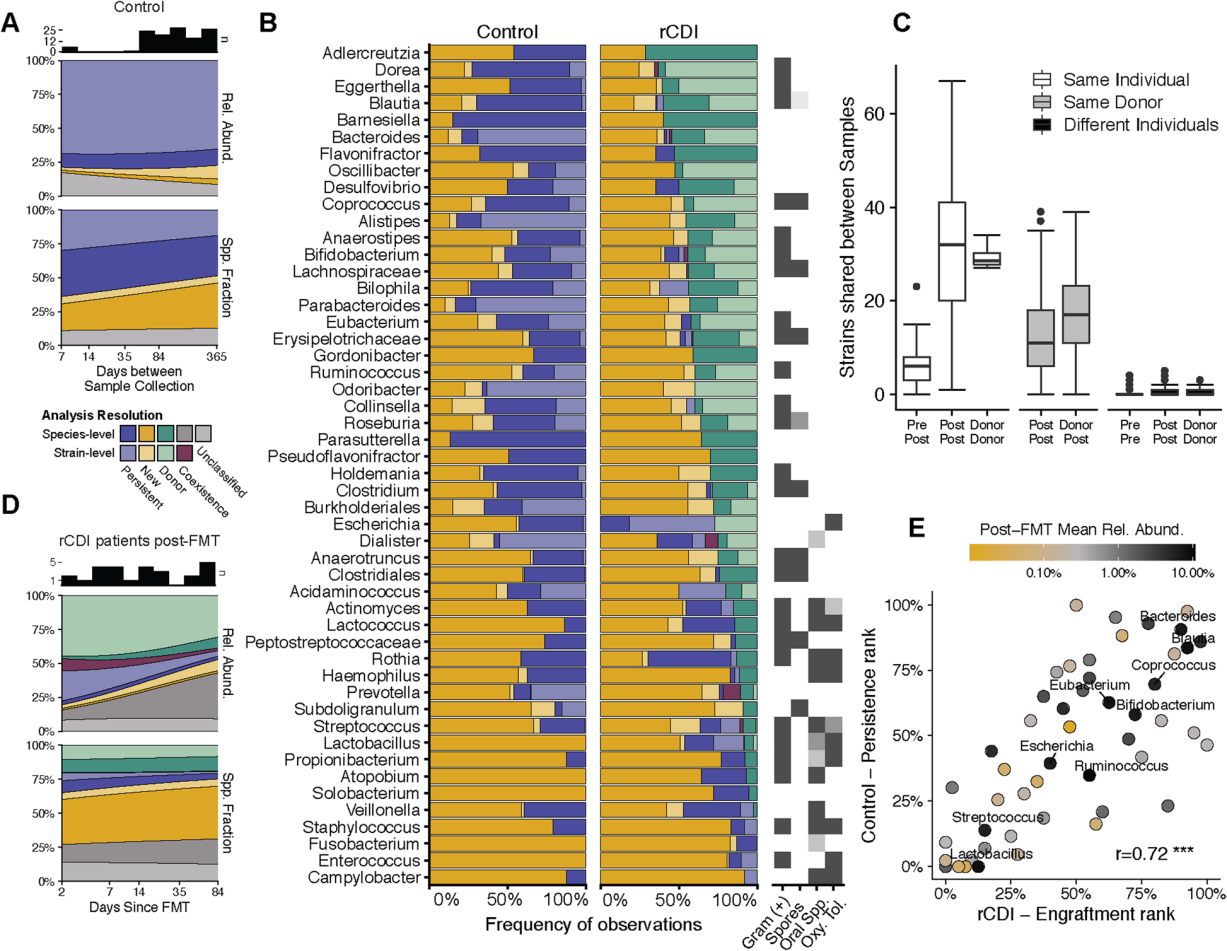
Identification of strain persistence and donor strain engraftment in healthy individuals and rCDI patients after FMT. **A** Longitudinal species and strain persistence in healthy adults from the reference (Control) cohort are shown as relative abundances of shared species and species fractions in 95 sample pairs from 59 individuals and modeled using binomial smoothing. Strain proportions are based on corresponding species. Species fractions indicate insufficient resolution for strain prediction. **B** Taxonomic variations in the frequency of species (dark blue), and strain (light blue) persistence in healthy individuals (*n* = 59) and FMT recipients (*n* = 19), and of donor species (dark green) and strain (light green) engraftment in post-FMT patients are shown, as summarized on the genus level for the 50 most prevalent genera (see Fig. S3 for species). Newly detected species and strains are shown in dark and light yellow, respectively. **C** Comparison of shared strain numbers between rCDI patients and donors. Distinct rCDI patients who received stool from the same donor share more strains than other post-FMT patients. **D** Donor-derived strains and species (exclusively shared with the donor but with insufficient resolution for strain prediction) account for large and stable relative abundances and species fractions in FMT-treated rCDI patients. Data for triads of successfully FMT-treated rCDI patients (*n* = 30) in reference to their pre-FMT (*n* = 19) and donor (*n* = 14) metagenomes are modeled across cases using binomial smoothing. **E** The frequencies of strain persistence in healthy individuals and of donor strain engraftment in rCDI patients after FMT are positively correlated at the genus level (Spearman’s *r* = 0.72, *p* < 1e−8), including for abundant members of the healthy adult fecal microbiota (see Fig. S5 for species-level comparison)

To study strain persistence in the fecal microbiota of healthy individuals, we used the reference cohort of 67 healthy adults described above and determined shared strains in sample pairs collected from the same individuals over periods of up to one year (Fig. 3A, see Fig. S2 for individual cases and samples). Contributions of temporally persistent strains that were shared between multiple samples from the same individual were relatively stable over time and comprised on average 22.6% ± 6.3 (mean ± sd) of all detected species in the later sample, which accounted for 73.1% ± 18.3 relative abundance. Strain persistence was detected with variable frequencies for different microbial genera (Fig. 3B) and species (Fig. S3). Based on the assignment of microbial species to different functional and lifestyle feature categories (see “Materials and methods” for details, Table S5), strain persistence was less frequent in oral and/or oxygen-tolerant genera (Fig. 3B) and species (Fig. S3).

To study strain persistence and engraftment in the course of FMT, we generated new metagenomic sequence data from our previously described cohort of FMT-treated rCDI patients [36, 37], which we combined with other available data [15] and applied SameStr to detect shared strains between pre- and post-FMT patients and post-FMT patients and donors (Fig. 3C, Table S7). Recipient and donor-derived species fractions and relative abundances in post-FMT patients were determined as being represented by shared strains between pre- and post-FMT patients or post-FMT patients and donors, respectively (Fig. 3D, see Fig. S4 for individual cases and samples). During the first week after FMT, both donor and recipient-derived strains contributed large relative abundances to the post-FMT microbiota (days 1–7: 42.5% ± 30.3 vs. 18.9% ± 22.3), but donor-derived microbiota fractions remained more stable over the following weeks and months, whereas recipient-derived microbiota fractions continuously decreased (days 70–84: 26.5% ± 21.9 vs. 4.9% ± 9.0). Donors and recipients before FMT frequently carried the same microbial species, but this rarely resulted in the detection of both recipient and donor-derived strains after FMT (Table S8). Consequently, coexisting recipient and donor strains from the same species accounted for only small and decreasing species fractions (0.46% ± 0.68) and relative abundances (5.19% ± 11.54) in post-FMT patients (Fig. 3D). Donor strain engraftment frequencies varied taxonomically and were less frequent in oral and/or oxygen-tolerant genera (Fig. 3B) and species (Fig. S3).

We next compared the healthy adult and FMT cohorts and found strains that frequently persisted in healthy individuals to belong to the same genera and species as donor strains that frequently engrafted in patients after FMT (Fig. 3E, see Fig. S5 for species comparison; Table S9-S10). Frequently persisting and engrafting genera included abundant (>5%) members of the healthy adult gut microbiota, such as *Bacteroides*, *Blautia*, *Coprococcus*, and *Eubacterium* (Fig. 3E), and similar observations were made at the species level (Fig. S5). Thus, FMT appears to specifically lead to the engraftment of persisting and abundant healthy gut microbiota members in rCDI patients.

### Identification of healthy individuals and FMT recipients and donors using shared strain profiles

**Fig. 4:**
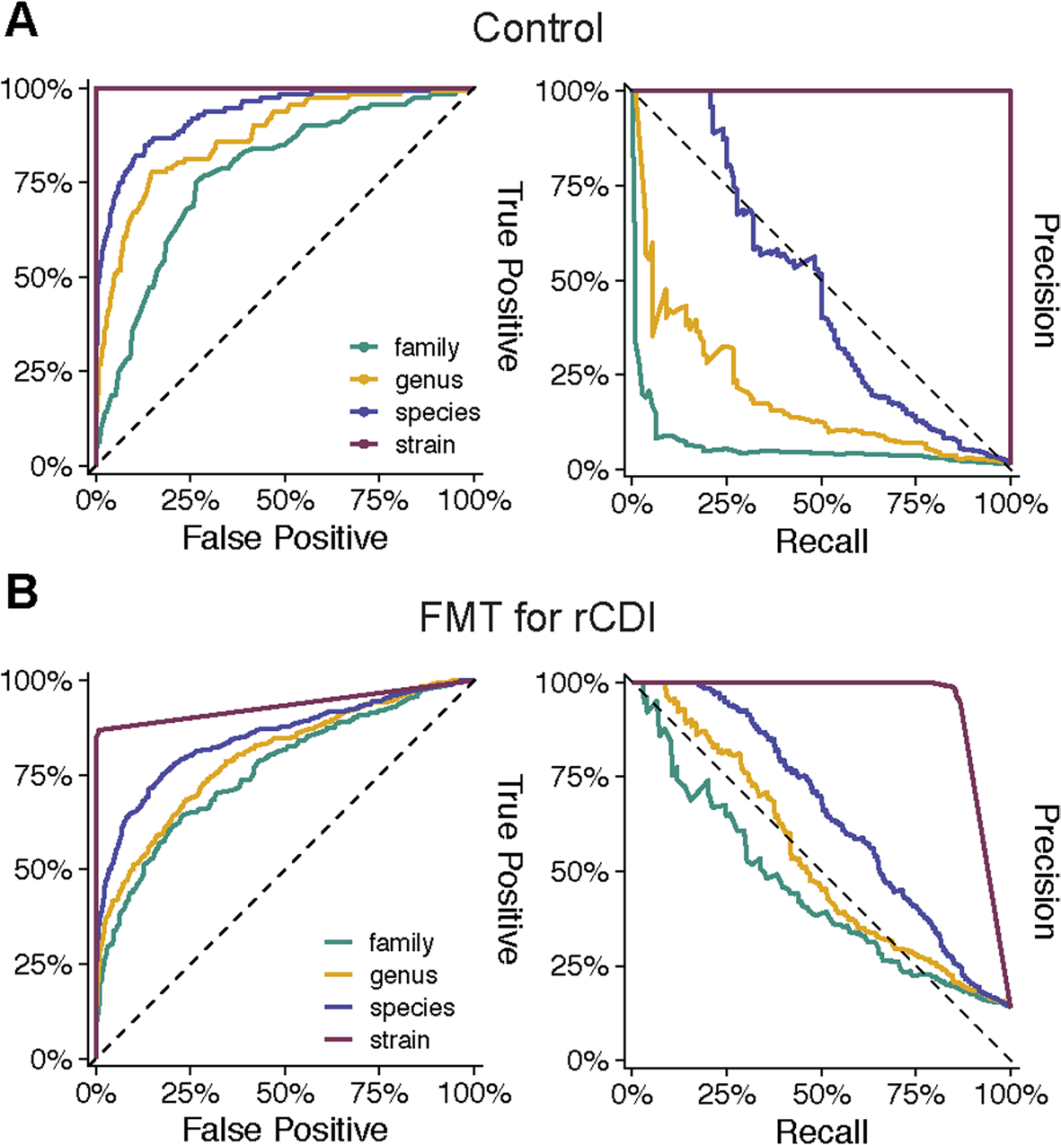
Identification of healthy individuals and FMT recipients and donors using shared strain profiles. Receiver-operating characteristic (ROC) and precision-recall (PR) curves of logistic regression classifiers demonstrate sensitive and accurate identification of (**A**) longitudinally collected sample pairs from the same healthy individuals (*n* = 112 from a total of *n* = 8120 sample pairs) and (**B**) related FMT patient and donor sample pairs (*n* = 580, including pre- and post-FMT patient samples, post-FMT patient and donor samples, and post-FMT patient samples that received FMT from the same donor, from a total of *n* = 4186 sample pairs)

The detection of species overlaps between the persisting core gut microbiota in healthy adults and the engrafted donor microbiota in rCDI patients after FMT, prompted us to test if individuals were identifiable based on shared strain profiles in fecal metagenomes. To this end, we first trained and tested a logistic regression classifier (60% / 40% data split for training and testing) to identify sample pairs from the same individuals in our healthy adult reference dataset, based on overlapping taxonomic microbiota compositions. Microbiota profiles at the family, genus, and species level were determined with MetaPhlAn2 and at the strain level with SameStr; total and shared taxa and strains were used as input for the classifier (Fig. 4A, Table S6). A perfect classification (auPR = 1, auROC = 1) of 8120 hold-out sample pairs (*n* = 112 sample pairs from the same individuals) was achieved with shared strain profiles, whereas shared family and genus profiles were insufficient (auPR ≤ 0.18, auROC ≤ 0.87) and even shared species profiles performed poorly (auPR = 0.47, auROC = 0.93). We next tested the same logistic regression classifier that was trained on healthy individuals for the identification of related sample pairs from the FMT cohort (*n* = 580 related compared to *n* = 3606 unrelated sample pairs), i.e., pre- and post-FMT samples from the same patients, corresponding post-FMT patient and donor samples, and post-FMT samples from different patients that received FMT from the same donor. Again, our classifier performed well using shared strain profiles as input (auPR = 0.94, auROC = 0.93) but not higher-level taxa profiles (Fig. 4B, Table S6). Thus, our findings demonstrate that the fecal microbiota of healthy adults harbors identifiable personal strain profiles, at least over periods of up to one year, which are transferable from donors to rCDI patients after FMT.

### Shared strain network analysis for the identification of mislabeled metagenomes

**Fig. 5:**
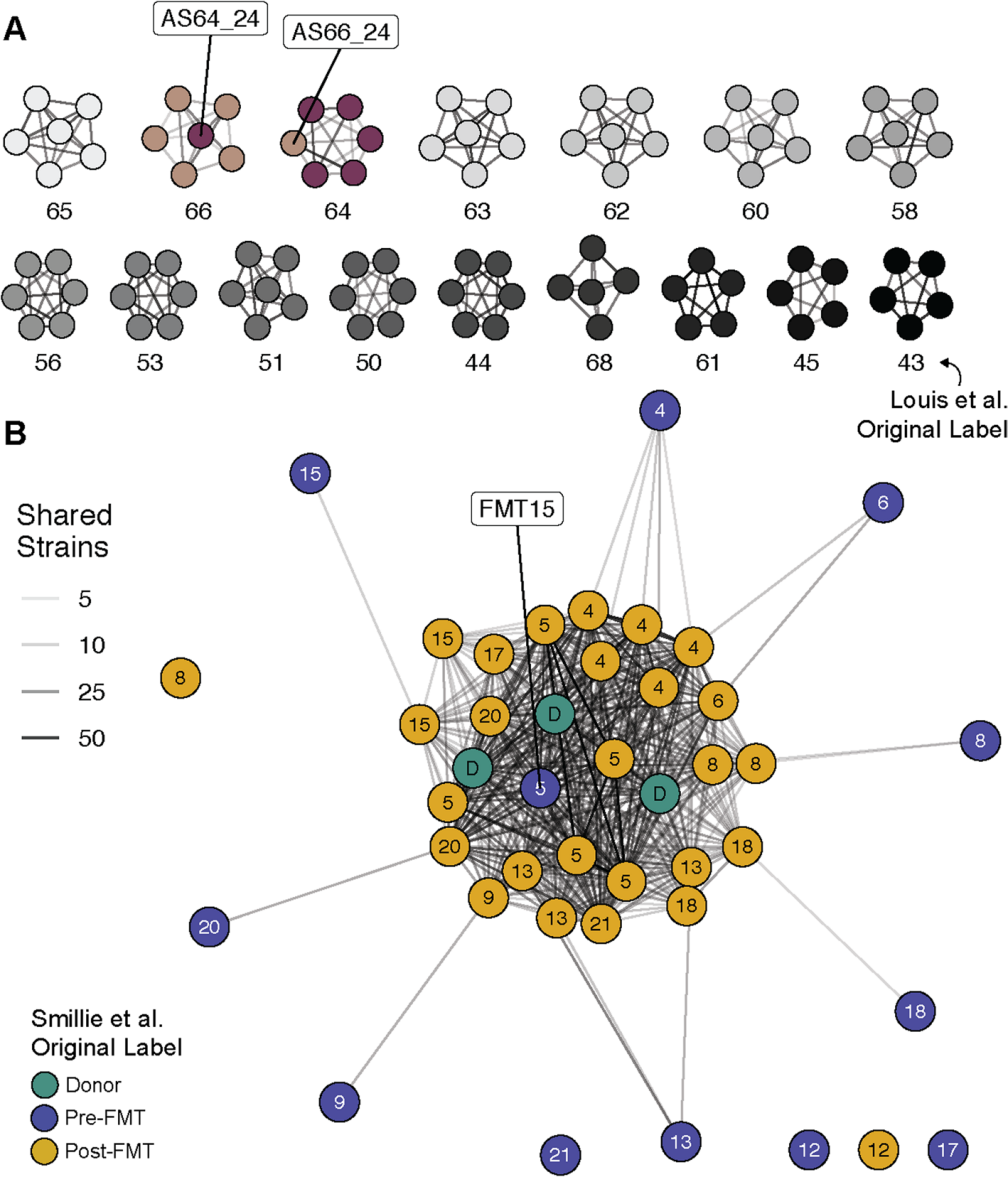
SameStr-based unsupervised strain sharing networks identify potentially mislabeled samples. Shared strain profiles were visualized as unsupervised networks with individual samples as nodes and shared strain numbers as edges. **A** These networks connect samples from Louis et al. [38] by individual, with the exception of two samples (AS64_24 and AS66_24) that appear to be mixed up. **B** In a case of multiple rCDI patients treated with FMT from the same donor [15], shared strains were detected between pre- (blue) and post-FMT (yellow) patient samples, as well as between post-FMT and donor (green) samples and among post-FMT samples. Pre-FMT samples did not share strains with donor samples, with the exception of FMT15, which shares (> 15) strains with all three donor samples and exhibits ɑ/β-diversity compositions that are comparable to other post-FMT samples (data not shown). As this sample was collected on the day of the FMT procedure, FMT15 could in fact represent a post-FMT sample that was accidentally mislabeled as a pre-FMT sample (Smillie, personal communication)

The published metagenomic sequence data used for this study included several samples that, while presenting with inconspicuous species-level taxonomic microbiota compositions, showed unexpected and inconsistent shared strain profiles that led to their removal from the analysis (Table S11). To illustrate these inconsistencies, shared strain profiles, as generated with SameStr, were visualized as unsupervised networks, which assigned related samples to distinct clusters linking, for example, samples from the same individual (Fig. 5A) or from FMT recipients and donors (Fig. 5B). However, in three cases > 2× more shared strains were detected between supposedly unrelated samples than between any of the other > 20,000 unrelated sample pairs from our dataset. In every case, suspicious sample pairs had been submitted as part of the same study and inconsistencies could be resolved by switching or changing sample labels (see Fig. 5 legend for details), suggesting sample mix-up or mislabeling. We have reported similar findings of potentially mislabeled samples in a meta-analysis of neonatal metagenomes [23], indicating that inconsistencies in public metagenomes might be common. Microbiota strain profiling with SameStr or equivalent tools could represent a viable strategy for the quality control of metagenomic sequence data from fecal microbiome projects.

## Discussion

We introduce SameStr as a new bioinformatic tool for the identification of shared microbial strains in metagenomic shotgun sequence data, which allows for the detection and quantification of strain persistence and transfer and improves our ability to track and understand subspecies population dynamics in complex microbiomes. In contrast to related methods that define strains more broadly and allow for the presence of the same strain in different, unrelated individuals [15, 16], SameStr applies a more conservative definition of strains as “unique” phylogenetic lineages that should only be shared by either temporally or physically related samples. It thereby affords the specificity to infer persistence or transmission from the detection of shared strains in distinct metagenomes. Recent fecal metagenomics-based epidemiological studies identified subspecies lineages or clades of, for example, *Prevotella copri* and *Ruminococcus gnavus* with widespread prevalence in the human population, which could be linked to dietary habits [39, 40] and host health background, i.e., inflammatory bowel disease [6], respectively. Strain-level microbiota profiling with SameStr provides the phylogenetic resolution to track even individual strains within these subspecies clades in the human population, illustrating new opportunities to shed light on the role of these and other microbiome members for human lifestyle adaptation and disease development.

Methodically, SameStr is related to the StrainPhlAn tool, as both use the taxon-specific marker gene database from MetaPhlAn [30] to identify and compare microbial species-specific single nucleotide variant profiles. However, SameStr’s approach to determine maximum variant profile similarities between metagenomic samples, including polymorphic alleles, demonstrates increased sensitivity for the detection of shared strains among multi-strain species populations, especially between subdominant strains. Dominant and secondary maternal strains of *Bifidobacterium* and *Bacteroides* species have been shown to compete for colonization in neonates after birth, contingent on their strain-specific carbohydrate-degrading capabilities [22], emphasizing the importance of considering multiple strains per species for the detection of strain sharing and microbial transfer. Other clinical use cases, specifically for SameStr’s conservative shared strain calls, could include, for example, the identification of strain sharing between the intestinal, reproductive, and/or urinary tract or bloodstream, which could be used to better characterize endogenous reservoirs of opportunistic pathogens and microbial translocation between human body niches as a cause of infection and disease [41, 42].

We applied SameStr to study strain persistence in the intestinal microbiota of healthy individuals, as well as strain persistence and engraftment in patients after fecal microbiota transplantation, using combined datasets from multiple studies, including healthy adults sampled over durations of up to one year and rCDI patients, sampled before and after FMT together with their donors. We detected strain persistence for many of the same bacterial taxa, such as *Bacteroides* species, as previously reported based on temporal single nucleotide polymorphism (SNP) stability [43] and strain-resolved species-specific MAGs [19] in fecal metagenomes from healthy individuals. Persistence has been negatively correlated to the genetic capacity for oxygen tolerance and sporulation before [19] and, based on comparative genome analyses, the loss of sporulation has been genetically linked to typical features of host-adaptation, such as genome reduction and metabolic specialization [44], confirming our functional predictions for species that are frequently represented by persisting strains, as well as our concept of a persisting core gut microbiota of strict anaerobe, non-spore-forming bacteria in the healthy human gut. We also identified a surprising taxonomic association between strain persistence and engraftment, as strains with a high persistence rate in healthy individuals belonged to the same bacterial species as donor strains with a high engraftment rate in rCDI patients after FMT. Given that persistence in the complex gut microbiomes of healthy individuals, as well as engraftment in the dysbiotic microbiomes of rCDI patients, requires strains to compete with other persisting, resident, and/or newly incoming strains, our analysis likely identified bacterial species of high ecological competitiveness and fitness. This is further supported by Hildebrand et al., who used the concept of tenacity to describe strain persistence in human individuals and described tenacious bacteria, such as *Bacteroides* species, as host-adapted, frequently dispersed by vertical transmission from mothers to infants, and most negatively affected by antibiotic perturbation [19]. In this context, the lack of sporulation genes in tenacious bacteria likely reflects an adaptive mechanism to increase persistence by avoiding excessive intra-species strain competition [19]. Using different methodologies, Watson et al. similarly concluded that FMT selects for high-fitness populations of the gut microbiome, based on the observation that a high prevalence of a microbial species in healthy individuals is more predictive for colonization success after FMT than a high relative abundance of the same species in a FMT donor [45]. Based on these considerations, the identification and characterization of stably persisting strains in healthy individuals could present a viable and more useful strategy to determine different constitutions of personalized, adapted core microbiomes of the human gut, than more commonly used β-diversity metrics based on species or higher-level taxon persistence. As key microbiome attributes, such as colonization resistance against pathogens [46] or resilience towards other perturbations [47] should be determined by the fitness of its core members, characterization of the persisting gut microbiota might constitute an ecological approach to define a healthy human gut microbiome [48].

Our analyses suggest additional practical applications for metagenomics strain profiling that extend previous concepts of microbiome-based forensic markers for personal identification [49]. Franzosa et al. identified combinations of taxonomic (operational taxonomic units and species), genomic (genome fragments), and functional (genes) markers as ‘metagenomic codes’ that could be used to match > 80% of fecal sample pairs that were collected over periods of 30-300 days from the same individuals [50]. Similarly, a majority of > 300 individuals could be identified in a mixed human cohort (auPR = 0.87, auROC = 0.95), using rare fecal metagenomic oligomers (k-mers of 18–30-bp length) [51]. Yet our shared strain-based personal identification method outperformed both previous attempts by demonstrating a 100% success rate for the detection of matching sample pairs (*n* = 112 from a total of 8120 sample pairs) from the same healthy individuals and, in addition, correctly identified most sample pairs from linked FMT donors and recipients (auPR = 0.94, auROC = 0.93 for *n* = 580 from a total of 4186 sample pairs). Standard practice for microbiome projects dictates the removal of human reads from metagenomic sequence data to de-identify samples before release. Our findings attest to the persistence and FMT-dependent transferability of personalized gut microbiome strain profiles and suggest that filtered public metagenomes retain personal information that could make study participants and FMT donors retrospectively identifiable.

The SameStr platform has a few limitations. First, as strains are identified based on SNV profiles in clade-specific marker genes, their detection is dependent on the underlying database and limited to previously described, sequenced, and comparatively analyzed taxa [30]. However, taxonomic assignments based on universal instead of species-specific marker genes, which are less dependent on available genome sequence information, can show discrepancies from established taxonomic systems [52], which could explain the increased taxonomic resolution and accuracy of SameStr’s taxonomic strain classifications compared to those from the StrainFinder tool. Moreover, SameStr can be easily adapted for use with updated (e.g., MetaPhlAn3, mpa_v30_CHOCOPhlAn_201901 [29]) or alternative, user-provided, marker sequences. Second, we developed SameStr specifically for the metagenome-based detection of strain sharing between fecal microbiomes. SameStr can be used to identify species that are represented by multiple strains, based on the detection of multiple alleles within a species-specific marker gene alignment of a single sample, with multi-strain species populations exhibiting ≥ 0.1% polymorphic positions of all detected alignment sites. However, it does not provide similar insights into strain population structures as related tools [15]. Third, in order to reliably detect strain-specific SNV profiles, SameStr required a sequencing depth of the genome corresponding to this strain of > 5-fold in our validation experiments, irrespective of whether this strain was the only representative or a minor component of a multi-strain species population. Assuming an average genome length of 2.5 Mbp and a metagenomic sequencing depth of 5 Gbp per sample (corresponding to 2000 genomes of average length), we estimate that SameStr is limited to the detection of shared or coexisting strains that make up at least 0.25% of all genomes in the metagenomic sample or 0.25% species relative abundance in case of single-strain species.

In conclusion, we present SameStr as a new bioinformatic tool for the species-specific, conservative identification of unique shared subspecies taxa in metagenomic shotgun sequence data, including subdominant members of multi-strain species populations. We demonstrate increased sensitivity, specificity, and taxonomic accuracy of detected strains in fecal metagenomes compared to related tools, which affords reliable detection of temporal strain persistence and transfer after fecal microbiota transplantation. We identify a persisting fecal core microbiota in healthy individuals, which taxonomically overlaps with the engrafted donor microbiota in rCDI patients after FMT, demonstrating the utility of SameStr to gain new insights into human gut microbiome stability and modulation. Application of this approach to other microbiome projects will improve our understanding of microbiome organization and function and should advance most areas of microbiome research.

## Materials and methods

### Study cohort

Metagenomic shotgun sequence data were generated from a previously published cohort of FMT-treated rCDI patients [36, 37]. The sample set included eight rCDI patient samples, collected 1–2 days before treatment, and eleven patient samples, collected between 1 week and up to 1 year after FMT. FMT was performed at Sinai Hospital of Baltimore, Baltimore, MD, USA, by single infusion of fecal filtrate from healthy donors into the jejunum and colon of rCDI patients. Study design, patient selection criteria, donor screening, infusion protocol, and sample collection have previously been reported [36].

### DNA isolation and sequencing

Metagenomic DNA extraction and sequencing of the 27 fecal samples was conducted at the Institute for Genome Sciences, University of Maryland School of Medicine. DNA was extracted from 0.25 g of stored fecal samples (− 80 °C), using the MoBio Microbiome kit automated on a Hamilton STAR robotic platform after a bead-beating step on a Qiagen TissueLyser II (20 Hz for 20 min) in 96 deep-well plates. Metagenomic libraries were constructed using the KAPA Hyper Prep (KAPA Biosystems/Roche, San Francisco, CA, USA) library preparation kit according to the manufacturer’s protocols. Sequencing was performed on the Illumina HiSeq 4000 platform to generate 150-bp paired-end reads.

### Published sequence data acquisition

Publicly available fecal metagenomic sequence data, longitudinally collected from healthy adult individuals, were obtained through curatedMetagenomicsData [35], including 202 metagenomes of 67 subjects (59 with known sampling days) from four different studies [38, 53–55]. Individuals were sampled at least twice within a year and had not reported medical conditions that would suggest extensive medication or strong microbiota perturbations between time points. For each subject, sequence data downloaded from the SRA were concatenated in case of multiple available accessions (Table S2). A total of 65 additional fecal metagenomes were obtained from 18 cases of FMT-treated rCDI patients who had not been treated with FMT before [15].

### Quality control and preprocessing of metagenomic sequencing data

All raw paired-end metagenomic sequence reads were quality-processed with Kneaddata v0.6.1 (KneadData Development Team, 2017) in order to trim sequence regions where base quality fell below Q20 within a 4-nucleotide sliding window and to remove reads that were truncated by more than 30% (SLIDINGWINDOW:4:20, MINLEN:70). To remove human sequence contamination, trimmed reads were mapped against the human genome (GRCh37/hg19) with Bowtie2 v2.2.3 [56]. Output files consisting of surviving paired and orphan reads were concatenated and used for further processing (Table S3).

### Metagenomic strain-level profiling with SameStr

The following individual analysis steps are part of the SameStr protocol to identify shared strains in metagenomic samples (Fig. 1):

1. *Taxonomic microbiota analysis.* Preprocessed sequence reads from each sample were mapped against the MetaPhlAn clade-specific marker gene database (db_v20, mpa_v20_m200) using MetaPhlAn2 v2.6.0 [57]. We additionally generated taxonomic profiles for rarefied data, which were subsampled to 5 M reads (after QC) per sample (seqtk v1.0) before processing with MetaPhlAn2, confirming representativeness of microbial communities as indicated by strong correlations of Shannon Index (diversity, vegan v2.5.7) between data.
2. *Detection of SNV profiles in marker gene alignments.* Using the SameStr tool, MetaPhlAn2 marker gene alignments were filtered for ≥ 90% sequence identity, a base call quality of Q20, and mapping length of 40 bp. The frequencies of all four nucleotides were tabulated with Samtools v0.1.19 [58] and kpileup v1.0 [15], retaining unmapped alignment sites as gap positions. Marker gene alignments were trimmed by 20 nucleotides at both ends, concatenated for each species, and combined from all samples. In order to address atypical vertical coverage and wrong base calls for each sample, alignment positions that diverged from the mean coverage by more than five standard deviations and alleles that were represented by < 10% of all mapped reads at this position were zeroed in the alignment.
3. *Determination of maximum variant profile similarity (MVS).* To consider individual strains from multi-strain species populations for the detection of shared strains, MVS were calculated between all species/alignment pairs *M*_i_ and *M*_j_ as the fraction of the sum of alignment positions with at least one shared allele *C*_allele_ divided by the sum of positions with coverage in both alignments *C*_cov_, where the vector of shared alleles *C*_allele_ was calculated as the pairwise Boolean product of 4 vectors of nucleotide counts at all positions between alignments *M*_i_ and *M*_j_. For consensus variant profile similarity (CVS) calculation, shared alleles were calculated as the pairwise Boolean product of a single vector representing the consensus sequence of the alignment at all positions between alignments *M*_i_ and *M*_j_.
4. *Comparison of reference genomes.* Species-specific marker gene regions were extracted from a total of 458 available genome sequences in the NCBI RefSeq and Genome databases from the 20 most abundant and prevalent species in our rCDI cohort (Table S4). For this, marker gene regions were extracted from reference genomes with a StrainPhlAn utility [17], based on BLASTn v2.6.0 comparisons, and used to generate multiple sequence alignments with MUSCLE v3.8.31 [59]. After removing gap positions, marker gene alignments were tabulated, concatenated, trimmed, and used to calculate the single-genome equivalent of MVS. MVS-based genome similarities were compared to average nucleotide identities (ANI), as calculated for entire genomes with FastANI v1.3 [31].
5. *Shared strain detection in distinct metagenomes.* Based on our reference genome comparison (Fig. 1C) and in agreement with previous reports [21], a MVS threshold of 99.9% was applied to detect shared strains that would be identified in related but not unrelated microbiomes. Shared strain predictions were additionally limited to sample pairs with at least 5000 overlapping alignment positions.
6. *Validation of SameStr on mock species populations.* Simulated shotgun sequence data were generated with ART read simulator v2.5.8 [60] and combined in various proportions to generate metagenomes from mock multi-strain species populations. Metagenomic paired-end sequence read error profiles were independently generated for each genome and simulation, using the Illumina HiSeq-20 error profile. For each species (Table S4), five reference genomes were randomly selected, including one target genome for shared strain detection and four other genomes to simulate a background noise of additional strains from the same species. Both the sequencing depths (fold coverage) of the target strain and its abundance relative to all other strains (noise coverage) were varied for each simulation. Marker gene alignments and comparisons for MVS or CVS calculation and shared strain detection were performed as described above.

### Classification of related and unrelated sample pairs

For the prediction of related samples (distinct samples from the same individual or connected samples from FMT donors and recipients) based on strain sharing, the number of detected and shared taxa between sample pairs from the healthy adult reference dataset were determined at the family, genus, species, or strain level with MetaPhlAn or SameStr, respectively, as described above. Data were divided into training and hold-out data (60%/40%) and shared taxon or strain fractions used to train simple logistic regression models (tidymodels v0.1.2). The classifier that was trained on strain persistence in healthy adults was then used to predict related sample pairs from the FMT cohorts. To assess the performance of the predictor, precision-recall (tidymodels v0.1.2) and receiver operating characteristic (ROC) curves were generated (tidymodels v0.1.2) and visualized (plotROC v2.2.1).

## Supplementary Information

**Additional file 1: Figure S1**. Computational Resource Requirements. (A) Total CPU time, (B) average CPU time per sample, and (C) average use of RAM by the Kneaddata, MetaPhlAn3 (mpa_v30_CHOCOPhlAn_201901), and SameStr programs during the processing of three datasets of different sizes. SameStr, on average, added 4.3 CPU minutes per sample to the computational effort of the entire workflow.

**Additional file 2: Figure S2**. Microbial Tracking across Individual Metagenomic Samples of Healthy Controls. Microbial tracking at the species (top) and strain level (bottom) in healthy controls. Healthy adults from the reference (Control) cohort harbor a core microbiota of persisting strains and species (insufficient sequencing depth for strain calls) shared between fecal metagenomes sampled up to one year apart.

**Additional file 3: Figure S3**. Predicting Donor Strain Engraftment in rCDI Recipients after FMT. The frequencies of species (dark blue) and strain (light blue) persistence in healthy individuals and rCDI recipients, and of donor species (dark green) and strain (light green) engraftment in post-FMT patients, differ between bacterial species, with retained recipient species and strains mostly being classified as oral and/or oxygen-tolerant species. Newly detected species and strains are shown in dark and light yellow, respectively.

**Additional file 4: Figure S4**. Microbial Tracking across Individual Metagenomic Samples of FMT-treated rCDI patients. Donor-derived strains and species (exclusively shared with donor but insufficient resolution for strain prediction) account for large and stable relative abundances across all post-FMT patient samples, whereas contributions of recipient-derived strains are comparatively smaller.

**Additional file 5: Figure S5**. Predicting Donor Strain Engraftment in rCDI Recipients after FMT. The same species that are represented by frequently persisting strains in healthy individuals are also represented by strains that frequently engraft from donors in rCDI patients after FMT and belong to species that have a high relative abundance in the healthy adult control cohort.

**Additional file 6: Table S1**. Sample and Case Metadata. **Table S2**. WGS Accession Identifiers. **Table S3**. WGS QC Data. **Table S4**. Reference Accessions. **Table S5**. Species Metadata. **Table S6**. Logistic Regression of (Un)related Sample Pairs at Taxonomic Levels. **Table S7**. Shared Species and Strains for Individual Cases. **Table S8**. Events with Competing Strains. **Table S9**. Strain Transmission Rates (per Species). **Table S10**. Strain Transmission Rates (per Genus). **Table S11**. Potential Sample Mislabellings.

## Data Availability

We implemented SameStr to facilitate the comparison of nucleotide variant profiles presented in this analysis. The program builds on previously published tools such as the concept of StrainPhlAn but extends the analysis of MetaPhlAn markers beyond a consensus-based approach by extracting all four nucleotide alleles from sequence alignments. Generated SNV-Profiles are in NumPy format and can be used as input for strain composition modeling and other analyses. The SameStr program and further documentation are available at GitHub: https://www.github.com/danielpodlesny/SameStr.git. R Markdown notebooks and additional code for generating figures are available at https://www.github.com/danielpodlesny/fmt_rcdi.git. Metagenomic sequence data are available from the European Nucleotide Archive under accession PRJEB39023.
