# Supplementary figures and images for: Metagenomic strain detection with SameStr: identification of a persisting core gut microbiota transferable by fecal transplantation
Source: Microbiome. 2022 Mar 25;10:53. doi: 10.1186/s40168-022-01251-w (PMC8951724; doi:10.1186/s40168-022-01251-w)

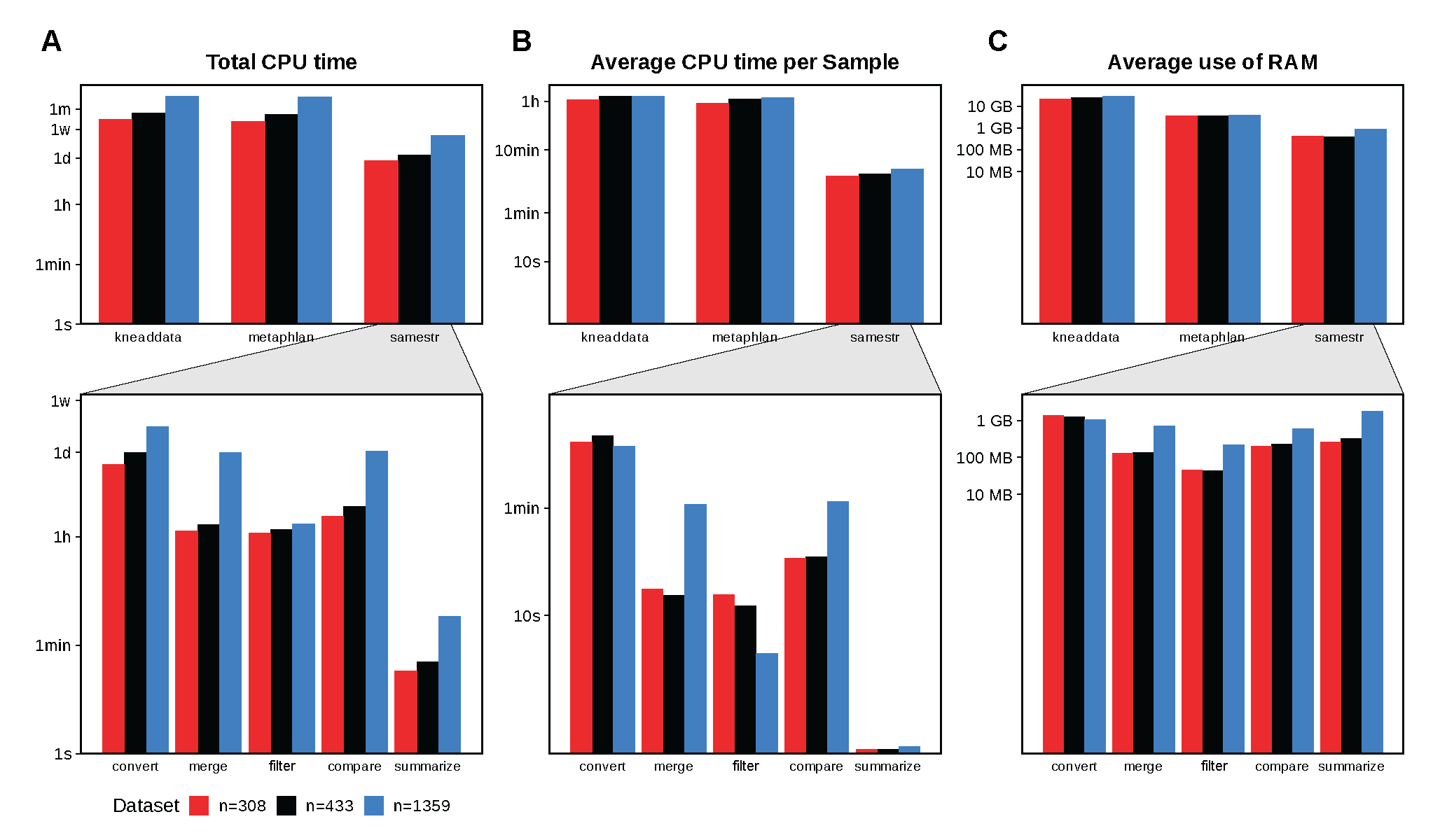

Supplement: Supplementary file 2 — Additional file 1: Figure S1. Computational Resource Requirements. (A) Total CPU time, (B) average CPU time per sample, and (C) average use of RAM by the Kneaddata, MetaPhlAn3 (mpa_v30_CHOCOPhlAn_201901), and SameStr programs during the processing of three datasets of different sizes. SameStr, on average, added 4.3 CPU minutes per sample to the computational effort of the entire workflow. [file 40168_2022_1251_MOESM2_ESM.png]

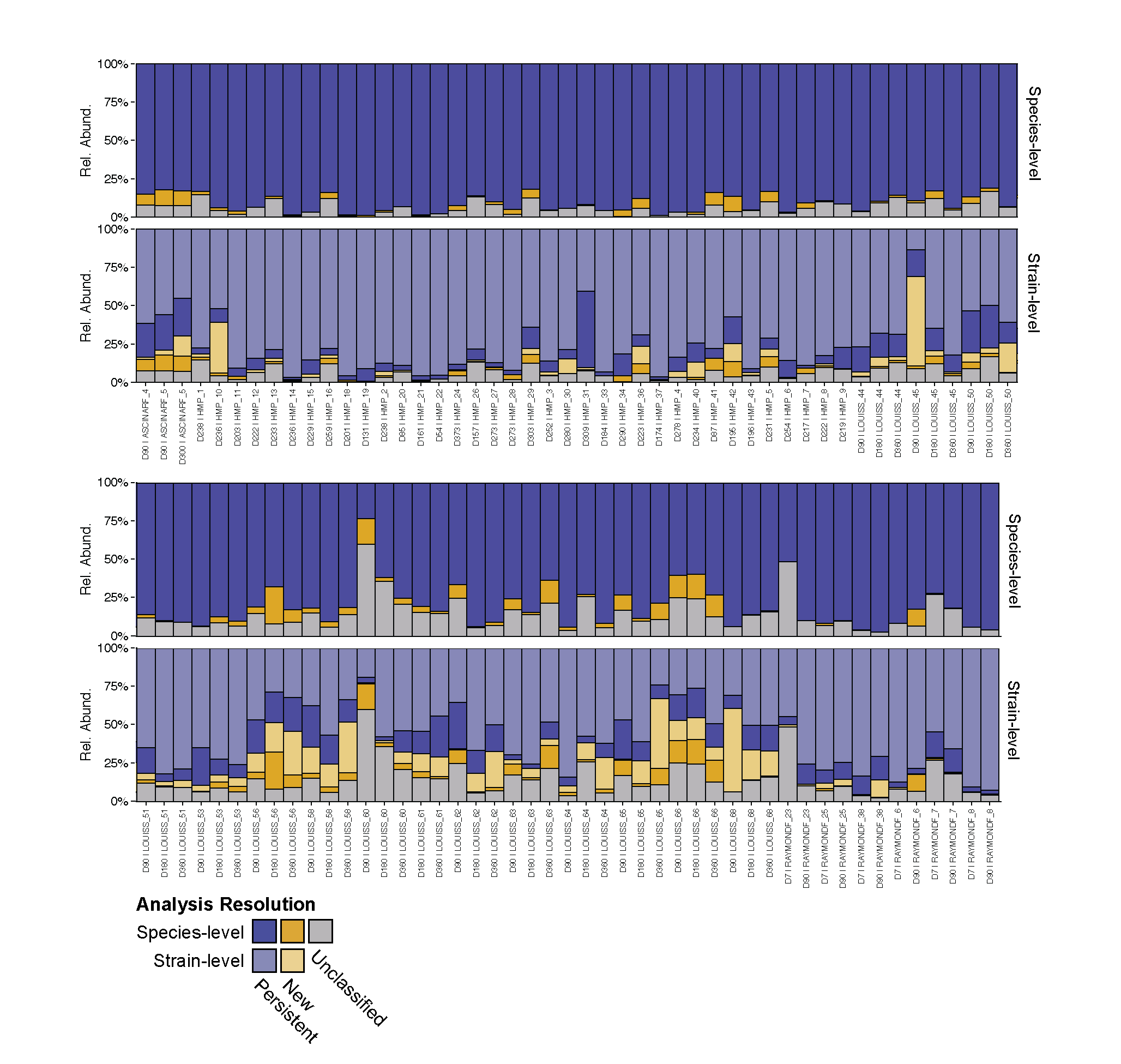

Supplement: Supplementary file 3 — Additional file 2: Figure S2. Microbial Tracking across Individual Metagenomic Samples of Healthy Controls. Microbial tracking at the species (top) and strain level (bottom) in healthy controls. Healthy adults from the reference (Control) cohort harbor a core microbiota of persisting strains and species (insufficient sequencing depth for strain calls) shared between fecal metagenomes sampled up to one year apart. [file 40168_2022_1251_MOESM3_ESM.png]

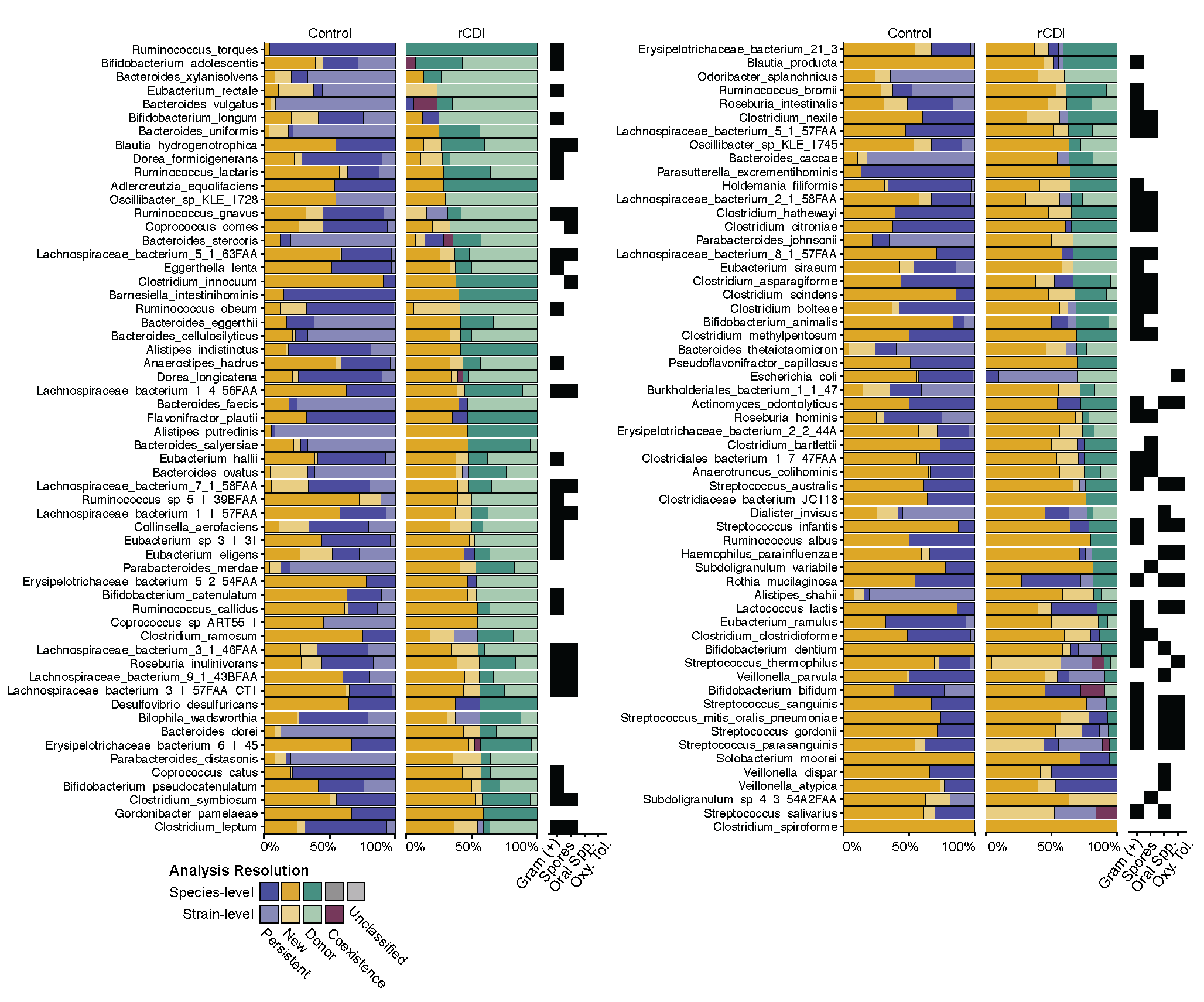

Supplement: Supplementary file 4 — Additional file 3: Figure S3. Predicting Donor Strain Engraftment in rCDI Recipients after FMT. The frequencies of species (dark blue) and strain (light blue) persistence in healthy individuals and rCDI recipients, and of donor species (dark green) and strain (light green) engraftment in post-FMT patients, differ between bacterial species, with retained recipient species and strains mostly being classified as oral and/or oxygen-tolerant species. Newly detected species and strains are shown in dark and light yellow, respectively. [file 40168_2022_1251_MOESM4_ESM.png]

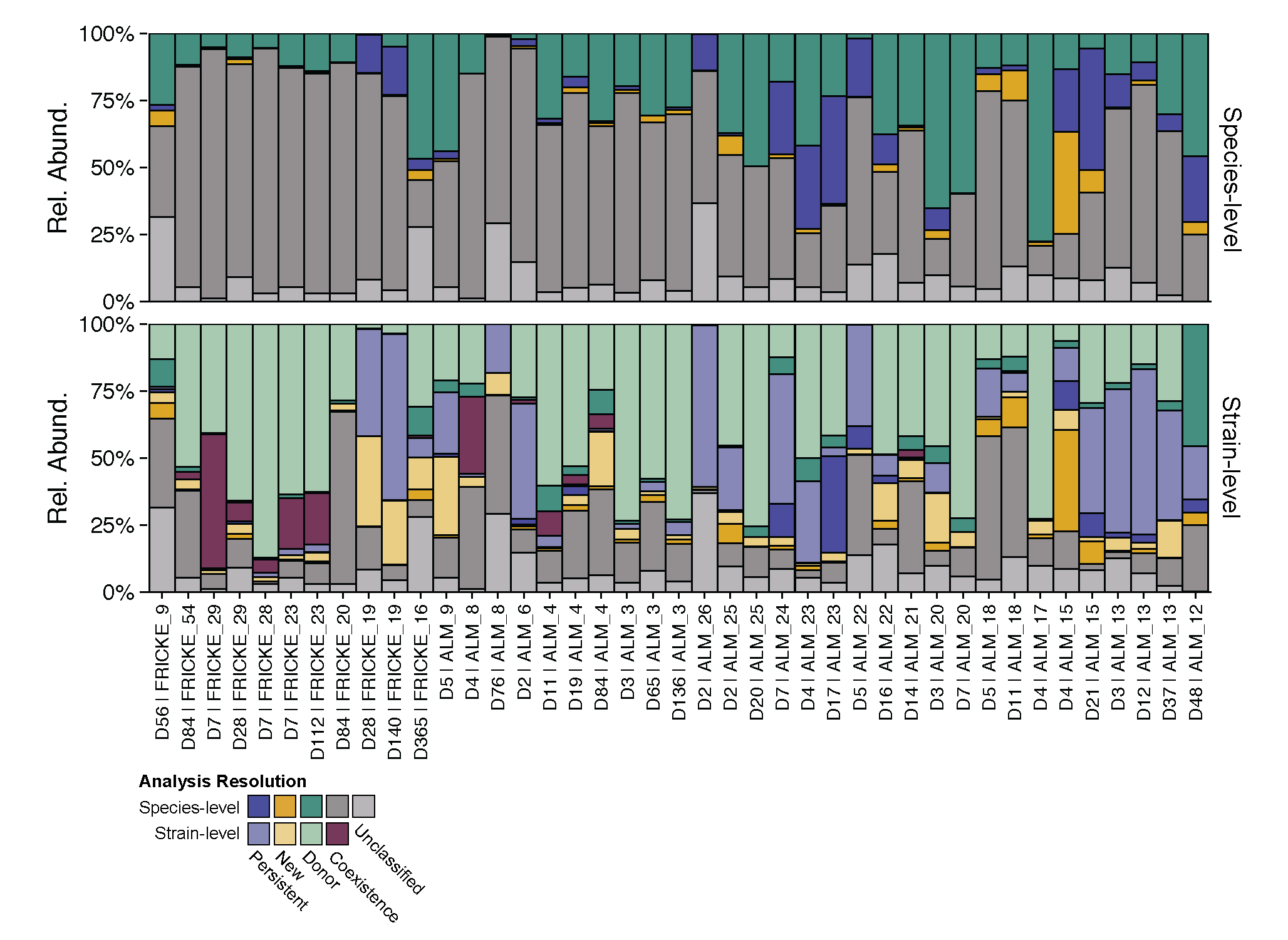

Supplement: Supplementary file 5 — Additional file 4: Figure S4. Microbial Tracking across Individual Metagenomic Samples of FMT-treated rCDI patients. Donor-derived strains and species (exclusively shared with donor but insufficient resolution for strain prediction) account for large and stable relative abundances across all post-FMT patient samples, whereas contributions of recipient-derived strains are comparatively smaller. [file 40168_2022_1251_MOESM5_ESM.png]

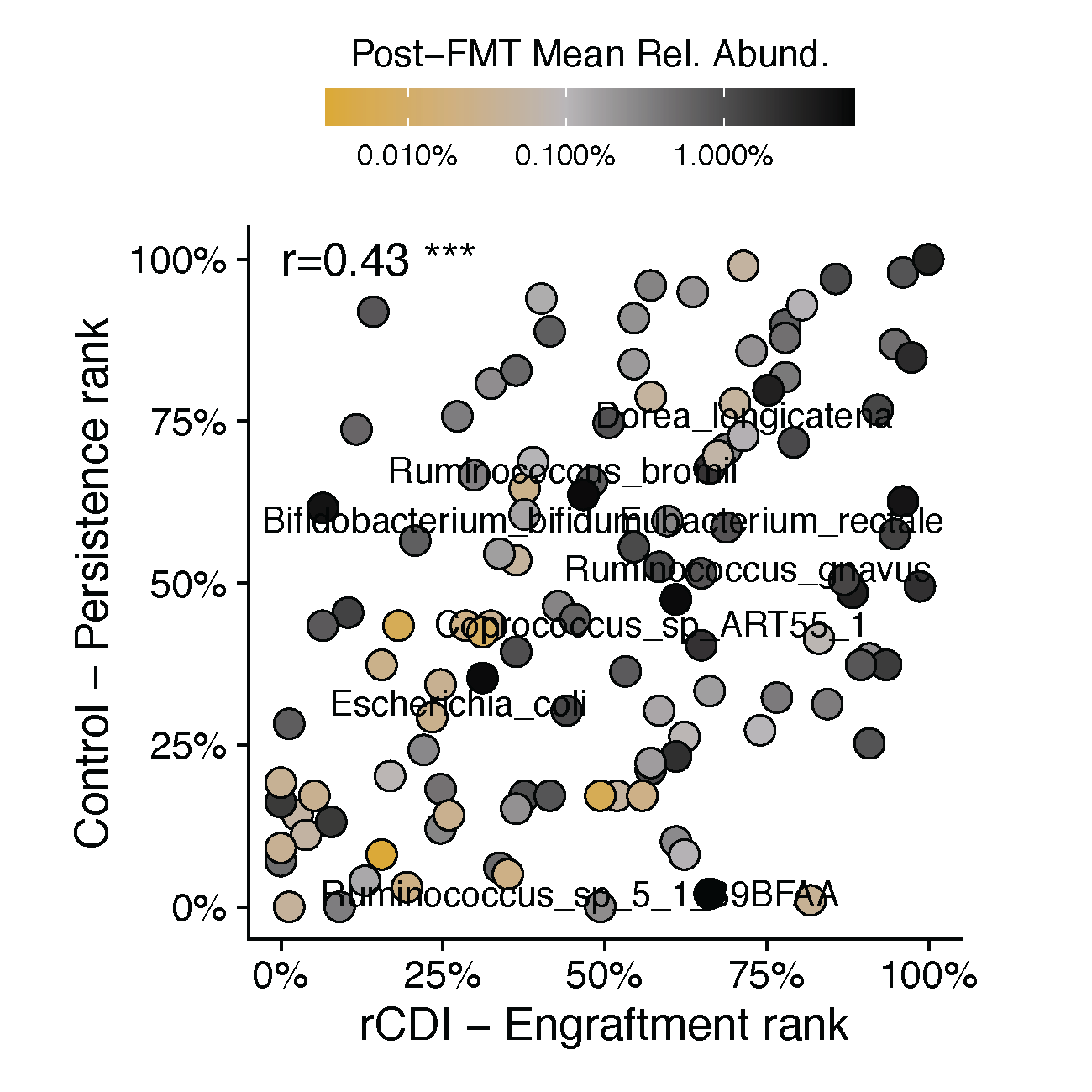

Supplement: Supplementary file 6 — Additional file 5: Figure S5. Predicting Donor Strain Engraftment in rCDI Recipients after FMT. The same species that are represented by frequently persisting strains in healthy individuals are also represented by strains that frequently engraft from donors in rCDI patients after FMT and belong to species that have a high relative abundance in the healthy adult control cohort. [file 40168_2022_1251_MOESM6_ESM.png]
